# Causal Learning From Predictive Modeling for Observational Data

**DOI:** 10.3389/fdata.2020.535976

**Published:** 2020-10-07

**Authors:** Nandini Ramanan, Sriraam Natarajan

**Affiliations:** Computer Science Department, University of Texas at Dallas, Dallas, TX, United States

**Keywords:** causal models, probabilistic learning, learning from data, structured causal models, causal Bayesian networks

## Abstract

We consider the problem of learning structured causal models from observational data. In this work, we use causal Bayesian networks to represent causal relationships among model variables. To this effect, we explore the use of two types of independencies—context-specific independence (CSI) and mutual independence (MI). We use CSI to identify the candidate set of causal relationships and then use MI to quantify their strengths and construct a causal model. We validate the learned models on benchmark networks and demonstrate the effectiveness when compared to some of the state-of-the-art Causal Bayesian Network Learning algorithms from observational Data.

## 1. Introduction

Given the recent success of machine learning, specifically deep learning, in several applications (Goodfellow et al., 2016), there is an increased interest in learning more explainable models including causal models.

Many researchers have attempted to develop methods to infer causality from observational data over for several years (Pearl, 1988b, 2000; Neapolitan et al., 2004). While there have been some notable contributions in the field demonstrating the plausibility of learning causality from non-experimental data (Granger, 1969; Sims, 1972; Pearl, 2000), learning structural causal models from observational data is still a challenge (Guo et al., 2019). Recent advances in the field of discovering causality has looked at learning Causal Bayesian Network (CBN). In this framework, causations among variables are represented with a Directed Acyclic Graph (DAG) (Pearl, 2000). The problem of learning a DAG from data is not computationally realistic as the number of possible DAGs grows exponentially with the number of nodes. This computational complexity has prevented the adaptation and application of causal discovery approaches to high dimensional datasets, with a few examples.

In this work, we consider the problem of full model learning of causal models from observational data. We are inspired by tasks in real-world where only limited knowledge could potentially be available and hence building a full causal model is not possible. Similarly, the data might be obtained before learning, making interventions particularly, hard. In such cases, learning a probabilistic causal model from data is preferred. However, this is a hard task with a larger number of variables. This is the problem we tackle in this paper—*how can we scale causal learning to a moderate number of features?*

To this effect, we build upon the success in using two sets of independencies for building causal models—that of mutual independencies (MI) (Janzing et al., 2015) and context specific independence (CSI) (Tikka et al., 2019). While MI can be used to quantify the strength of the causal relationships, CSI has been used for causal identifiability. We employ these in the context of learning from data. We aim to learn a causal model by first learning probabilistic dependencies that can identify CSI. We then adopt a heuristic measure to remove and re-orient the edges of the probabilistic graphical model. We employ MI and heuristics to guide the search. The net result as we show empirically is a causal model. This is particularly important as scaling causal learning to large problems without interventions or bias is a significantly challenging task.

Specifically, we leverage the success of dependency networks (DN) (Heckerman et al., 2000; Neville and Jensen, 2007; Natarajan et al., 2012) for learning with large data sets. Recall that a DN is a probabilistic graphical model that approximates the joint distribution using a product of conditionals. Hence, compared to a Bayesian Network (BN) these are uninterpretable and more importantly, approximate. However, their key advantage is that since they are products of conditionals, the conditionals can be learned in parallel and can be scaled to very large data sets.

To scale causal model learning, we first learn a DN. To perform this, we learn a single (probabilistic) tree for every variable, then we identify and remove cycles from this DN. We consider mutual information employed in causal models to score and remove the edges. In addition, we detect and remove cycles from the DN, if any. Contrary to popular intuition, we employ two levels of learning to uncover a causal model—first is on learning a DN using trees and the second is on learning a causal model employing heuristics measures. Our evaluations on the two synthetic and one real benchmark causal data sets demonstrate the utility of such an approach. While we present quantitative metrics, qualitatively, the edges that are learned in this model uncover interesting findings. In addition, we compare the proposed approach to three other state-of-the-art causal learning methods employed on just the non-experimental data. Our results demonstrate that we obtain most of the causal links on large problems in order-of-magnitude fewer operations than most causal approaches.

We make a few crucial contributions—we present the first causal learning approach that leverages progress in probabilistic methods toward learning from data. We develop heuristics on breaking the cycles and orienting the edges based on the causal modeling research. We learn a causal model on two synthetic and one real benchmark causal data sets and compare with ground truth network to understand the robustness of our approach. We also demonstrate the efficacy and efficiency of the approach on standard benchmark data sets compared to other state-of-the-art constrained based methods in the literature. Our proposed approach opens the door for a domain expert to interactively guide the causal model learner to a better model thus allowing a hybrid method for causal models.

The rest of the paper proceeds as follows: after reviewing the related work on BN, followed by the discussion of some notable work in constrained based methods for learning CBN, we provide the background on DN learning. Next, we present our algorithm and provide intuitions on its functionality. We discuss the motivation of this work, that of the three benchmark data sets which are used to learn the joint causal model over the factors. Then we present the empirical evaluations on the two synthetic benchmark causal data sets and one real data set by comparing our algorithm with other commonly used Causal learning approaches as well as the ground truth. Finally, we conclude by outlining potentially interesting future directions.

## 2. Background and Related Work

We first introduce Bayesian networks and dependency networks and certain concepts which build the foundation for innovations in CBN learning.

### 2.1. Bayesian Network

A Bayesian network (BN) is a directed acyclic graph *G* = 〈**V**, **E**〉 whose nodes **V** represent random variables and edges **E** represent the conditional influences among the variables. A BN encodes factored joint representation as, *P*(**V**) = ∏_*i*_*P*(*V*_*i*_∣**Pa**(*V*_*i*_)), where **Pa**(*V*_*i*_) is the parent set of the variable *X*_*i*_. It is well-known that full model learning of a BN is computationally intensive, as it involves repeated probabilistic inference inside parameter estimation which in turn is performed in each step of structure search (Chickering, 1996). Therefore, much of the research has focused on approximate, local search algorithms that are generally broadly classified as constraint-based and score-based.

In constraint-based methods, we learn a BN which is consistent with conditional independencies inferred from data (Spirtes et al., 2000). By contrast, score-based methods search through the space of structures, and find the structure with the highest score (Heckerman et al., 1995; Friedman et al., 1999). Hybrid learning approaches combine the advantages of both approaches; for example, using constraint-based techniques to estimate the network skeleton, and using score-based techniques to identify the set of edge orientations that best fit the data (Tsamardinos et al., 2006).

Our work is inspired by and can be considered as extending constraint-based methods which have been discussed extensively in the context of causal structure discovery.

### 2.2. Constraint-Based Algorithms

Constraint-based methods for learning causal structure from just the observational data typically use tests for conditional independencies to identify the causal links that exist in the data.

Following three assumptions are employed to connect the underlying causations that are not perceived directly to observable probabilistic dependencies:

- The **Causal Markov Assumption** states that every variable in a causal DAG *G*_*c*_ is (probabilistically) independent of all other variables if all its parents are observed.
- The **Faithfulness Assumption** states that a causal DAG *G*_*c*_ and probability distribution *P* are faithful to one another iff the only conditional independencies in *P* are those entailed by the *Causal Markov Condition* on *G*_*c*_.
- The **Causal Sufficiency Assumption** that there doesn't exist a common unobserved cause of one or more nodes in the domain (no hidden cause).

The *Causal Markov Assumption* produces a set of (conditional and unconditional) probabilistic independencies from a causal graph, and the *Faithfulness Assumption* ensures that all of the probabilistic independencies in the distribution are entailed by the causal Markov condition. The above stated three assumptions together ensure that causal DAG *G*_*c*_ meets the *Minimality Condition*. The minimality condition ensures that there exists no proper subgraph of the true causal DAG *G*_*c*_ that can satisfy the causal Markov assumption as well as produce the same probability distribution (Zhang, 2008).

Consequently, the constraint-based methods for causal discovery are both sound and complete given perfect (noise-free) data (Spirtes and Glymour, 1991; Zhang, 2008; Colombo and Maathuis, 2014). The well-known PC algorithm assumes no latent variables and learns a BN consistent with conditional independencies inferred from data (Spirtes et al., 1993; Margaritis and Thrun, 2000). PC and a related algorithm FCI (Spirtes et al., 2000) take a global approach to causal discovery by learning a network to model the joint distribution. The FCI algorithm in addition can model latent confounders. However, they require searching over exponential space of possible causal structures. This restricts their adaptation to high-dimensional data (Silander and Myllymaki, 2012). Consequently, there are extensions of FCI, RFCI (Colombo et al., 2012) that improve the efficiency at the cost of model quality.

PC algorithm is heavily variable order dependent, i.e., if the order of the variables changes during learning, the resultant causal Bayesian network could potentially change. Stable-PC (Colombo and Maathuis, 2012) is a modified version of the PC algorithm that queries all the neighbors of each node while computing CI tests and yields order-independent skeletons. Modified PC is efficient enough to handle large sets of variables, at the cost of not being provably sound and complete (Coumans et al., 2017). To overcome the inefficiency of computing CI test between all pairs of variables, algorithms to uncover only local causal relationships between a specific target node and its neighbors have been developed (Margaritis and Thrun, 2000; Aliferis et al., 2003; Ramsey et al., 2017). A well-known work in this line of research is Grow Shrinkage algorithm (GS) (Margaritis and Thrun, 2000). GS is based on the idea that the Markov blanket includes all the nodes that contain the information about the current node being tested. Although the PC algorithm and the GS algorithm have had a major impact in this area of research, GS is still exponential in the size of the Markov blanket.

Following the success of GS, several methods, such as IAMB (Tsamardinos et al., 2003) and its variants (Yaramakala and Margaritis, 2005) have been developed for the induction of CBNs by identifying the neighborhood of each node. Unlike PC and FCI, a well-known algorithm called Greedy Equivalence Search (GES) (Meek, 1995) begins with an empty graph and adds and removes edges iteratively. The GES algorithm falls broadly under a score-and-search procedure, that searches over equivalence classes of DAG and scores them (Chickering, 2002a,b). Although GES works well with moderate number of nodes, the space of equivalence classes is exponential in the number of nodes (Gillispie and Perlman, 2013). The Greedy Fast Causal Inference (GFCI) combines the benefit of GES (to learn the network) and FCI (to prune unnecessary edges as well as orient the edges) (Ogarrio et al., 2016). Meanwhile, there has also been more and more evidence demonstrating the possibility of discovering causal relationships by combining both experimental and observational data (Cooper and Yoo, 2013; Hauser and Bühlmann, 2015; Meinshausen et al., 2016). Other notable direction involves learning from mixed data types (continuous and discrete variables) (Andrews et al., 2018; Tsagris et al., 2018). In principle, our approach can be naturally adapted to handle mixed variable types, as long as an appropriate conditional independence test is employed. However, we note this as a future direction.

Our approach can be seen as scaling such methods to large observational data by potentially identifying a cyclic dependency network that can then be transformed into a causal graph. As mentioned earlier, we move away from the data-driven independency tests and consider model-based independency tests which could allow us to scale to potentially large data sets. We hypothesize that learning such a dependency network is scalable thus reducing the complexity of causality search.

### 2.3. Dependency Networks

Dependency Networks (DN) (Heckerman et al., 2000), another directed model is similar to a BN, except that the associated network structure need not be acyclic. That is to say, unlike a BN, a DN permits cycles. A DN encodes conditional independence constraints such that each node is independent of all other nodes, given its parents (Heckerman et al., 2000). Therefore, they approximate the joint distribution over the variables as a product of conditionals thus allowing for cycles. These conditionals can be learned locally, resulting in significant efficiency gains over other exact models, i.e., **P**(**V**) = ∏_*V*∈**V**_**P**(*V*|**Pa**(*V*)), where **Pa**(*V*) indicates the parent set of the target variable *V*. Since they are approximate [unlike standard Bayes Nets (BNs)], Gibbs sampling is typically used to recover the joint distribution; this approach is, however, very slow even in reasonably-sized domains. In summary, learning DNs is scalable and efficient, especially for larger data sets, but BNs are preferable for inference, interpretation, discovery and analysis. Recall that our goal is to discover causal relationships between variables. In order to develop an approach for this motivating application, we propose an algorithm for learning a BN from DN, that can scale to a large number of variables.

## 3. Exploiting Context-Specific Independencies for Learning Causal Models

Given the necessary background, we now present our learning algorithm for learning causal models from data. Our method is purely data-driven—extending this work to exploit domain expertise is an important immediate future direction. However, it must be noted that incorporating human advice as inductive bias, search constraints and/or orientation knowledge is natural in our framework. In this work, we assume that only the data and (if available) some ordering over the variables as inductive bias is provided.

We use bold capital letters to denote sets (e.g., **V**) and plain capital letters to denote set members (e.g., *V*_*i*_ ∈ **V**). Using this convention, we denote the set of variables as **V**. The goal of our algorithm is to learn the joint distribution over all the variables (features and the target) that models causality. Given that there is no additional input, it is quite possible that the joint distribution that is purely learned from data may not result in a causal model, i.e., the learned network is a general Bayes net (BN) instead of a causal Bayes net (CBN). To evaluate this, we verify the learned model on a few benchmarks to demonstrate the efficacy of the approach. Beyond empirical evaluations, we provide some theoretical insights on why the learned model is causal. Before explaining the procedure, let us formally define the learning task.

**Given:** Data, $\text{D}={\langle \langle V_{1}^{i},\ldots ,V_{n}^{i}\rangle \rangle }_{i=1}^{m}$, where *n* is the number of variables, *m* is the number of examples, **V** is the set of variables,

**To Do:** Learn a causal joint distribution, *P*(**V**), i.e., a causal BN 〈**V**, **E**〉, where **E** is the set of edges in the causal BN.

**Figure 1:**
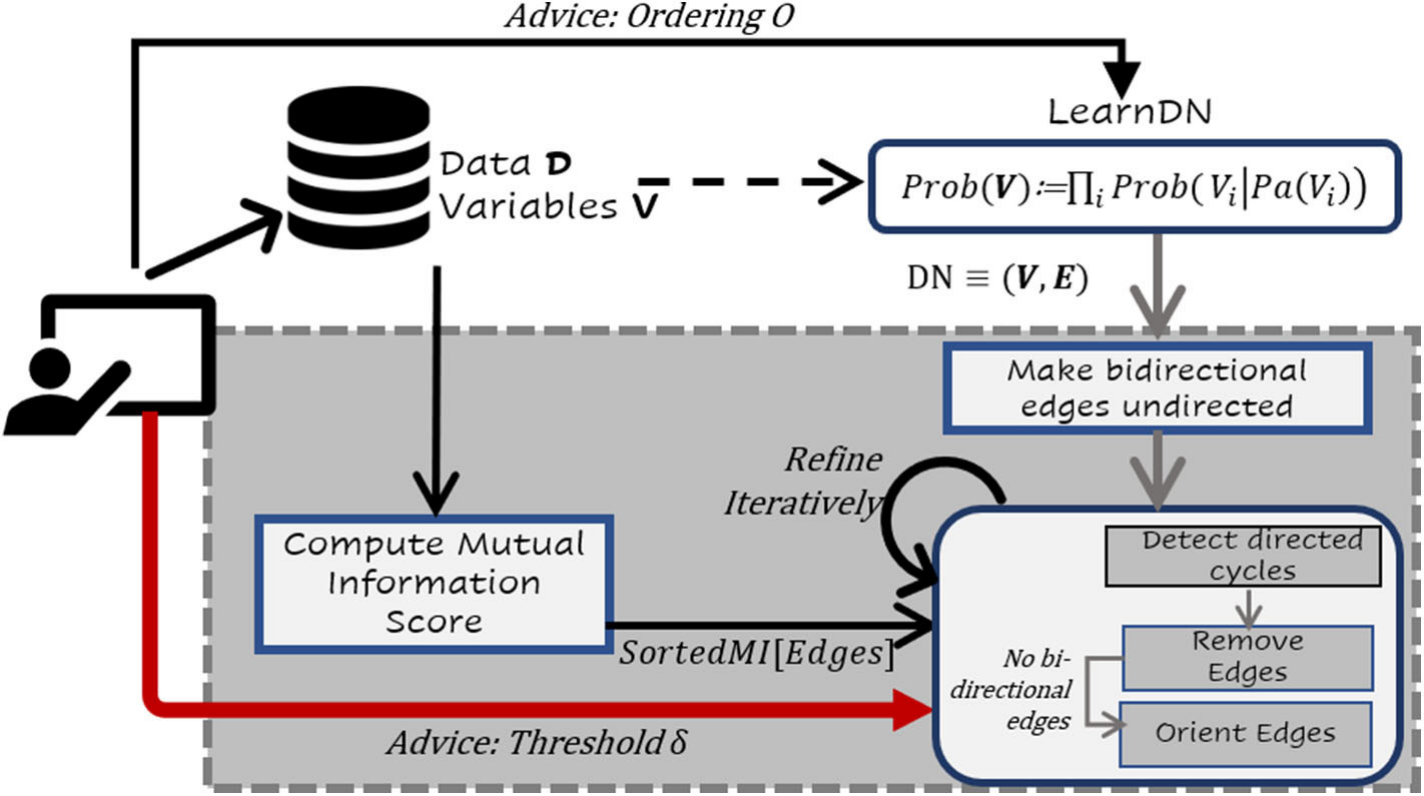
Flow Chart of the proposed framework. Given data *D* with *V* variables, a dependency network *DN* ≡ (**V**, **E**) is learnt on entire data. Learn a dependency network where each conditional is a decision tree of small depth. Recollect that resultant *DN* may have bidirectional edges between nodes. All the bidirected edges in the *DN* are converted to undirected edges (if any). For all variables with edges in between them in *DN*, mutual independence scores between them are computed. We loop through all the cycles in *DN*, such that the shortest cycles from the *DN* are first identified and the appropriate edges are removed based on MI less than the threshold δ. Our framework also allows for an expert to provide the predefined threshold δ. The process is repeated until there are no more directed cycles. Finally, the undirected edges are oriented based on MI while preserving acyclicity.

One of the challenges with standard BN learners and certainly CBN learners is that of scale. When the number of variables is large (as in the real benchmark data set), many structure learning algorithms do not scale viably. Hence, we propose a hybrid approach that combines the salient features of both search and score, namely the ability to perform local search effectively with the ability of constraint-based methods to potentially identify causal models. More precisely, our algorithm performs three steps: learning a dependency network from data, detect the cycles and then remove the edges that are mutually independent. This process is illustrated in Figure 1. The overall intuition behind this approach is fairly simple: use a scalable algorithm to handle a large number of variables and learn a dense model quickly. Since this learned model could potentially (and in practice) contain many cycles, we detect and remove edges based on mutual information. We then orient the edges ensuring acyclicity. Given that previous literature has demonstrated that an information-theoretic measure based on mutual information between two variables *X* and *Y* can be used as a reliable measure for quantifying the strength of an arc *X* → *Y* (Solo, 2008; Weichwald et al., 2014; Janzing et al., 2015), we use CSI and MI to establish the causal relationships.

We now describe each of these steps in detail before presenting the high-level algorithm.

### 3.1. Learning Context-Specific Independences

The first step of our learning algorithm is to learn distributions of the form *P*(*V*_*i*_|**V**\*V*_*i*_), i.e., a conditional for a variable given all the other variables in the data. To this effect, we employ the intuition that a structured representation of a conditional probability table (CPT), such as a tree can be used inside probabilistic models to capture *context-specific independence* (CSI) (Boutilier et al., 1996). Specifically, we learn a single probability tree for each variable *V*_*i*_ given all the other variables in the data. The tree CPDs can capture *context specific independence* based on regularities in the CPTs of a node. Tree CPD for a variable is a rooted tree with each interior node representing tests on parent vertices and leaf nodes have the probability conditioned on particular configurations along the path from the root to leaf. The key idea here is that each tree can capture the CSI that exists between the variable's parents and the target variable conditioned on the values of some of the other parents. This is an important step as *it has been recently demonstrated that CSI can be used for identifying causal effects by* Tikka et al. (2019). While their work derives the calculus for identifying the causal relationships, we go further in employing the use of CSI in larger data sets. Further, our finally learned network can be considered as a special case of the structural causal model proposed by Tikka et al. where the structured representations (trees) are used to model the CSIs and the edges of the graphical model are aligned using information-theoretic measures.

To learn CSI at every variable, we employ the notion of DNs. Recall that a DN is a (potentially cyclic) graphical model that approximates the joint distribution as a product of conditionals. To learn such a DN, we iterate through every variable and learn a (probabilistic) decision tree for each variable given all the other variables, i.e., the goal is to learn *P*(*V*_*i*_|**V** \ *V*_*i*_) for each *i* where each conditional is modeling using a probabilistic tree. We observe that in this step, one could provide an important domain knowledge—*ordering between the variables*. This variable ordering can be used to construct expert guided causal model which introduces CSIs that satisfies the ordering constraints. As shown by Tikka et al. (2019), the conditional distributions induced using these CSIs can be effectively employed in identifying do calculus.

The advantage of this approach is that it learns the qualitative relationships (structure) and quantitative influences (parameters) simultaneously. The structure is simply the set of all the variables appearing in the tree and the parameters are the distributions at the leaves which can be reused in later stages. The other advantage is that the approach is that it is easily parallelizable and scalable. Thus, our method can be viewed as one that could scale up learning of causal models to real large data sets. The third advantage of the approach is that being a separate step, this can be integrated with other causal search methods, such as the one proposed by Tikka et al. Exploring these connections is an interesting future direction.

Let us denote the conditionals learned over all the variables (potentially given some order) as *DN*, the dependency network induced from the data. In most cases, this DN contains cycles since these conditionals are learned independent of each other. This can be an advantage and a disadvantage. The advantage is its efficiency as the costly step of checking for acyclicity can be avoided during learning and a disadvantage since it is an approximate model. Shorter cycles can result in larger approximations (Heckerman et al., 2000). After learning this *DN*, we perform an additional step. We convert edges of the form *X* ← *Y* and *X* → *Y* to *X* − − *Y*. This is similar to the PC algorithm (Spirtes et al., 2000) in that strong correlation between two variables are considered as undirected and will be oriented in the final step of our algorithm. Next, we convert the DN to an intermediate CBN with potential undirected edges.

### 3.2. Detecting and Removing Cycles

To convert the DN to a CBN, the first step is to detect and remove cycles. A naïve approach to deleting edges would be: search for an edge, remove it, check for acyclicity and log-likelihood (Hulten et al., 2003). The key limitation of this approach is that the resulting model is not necessarily causal. The use of log-likelihood does improve the training performance but does not guarantee causality. Hence, inspired by the research in information-theoretic approaches to causality (Solo, 2008; Weichwald et al., 2014; Janzing et al., 2015), we employ mutual information for identifying the edges.

For detecting cycles, several methods exist (Kahn, 1962) including topological sorting. Any of these methods would be compatible with our learning algorithm. For the purposes of our data sets, we employ depth-first search (DFS). One key aspect of our DFS is that we identify short cycles. Recall that DN approximates a joint distribution as a product of conditionals.

$$\begin{array}{l} P\left(V_{1},...,V_{n}\right)\approx \underset{i}{\prod }P\left(V_{i}|\text{V}\backslash V_{i}\right) \end{array}$$

The theoretical analysis of the approximation is based on the inference algorithm, specifically Gibbs sampling and on the size of the data. In simple terms, if the Gibbs sampler converges on a large data set, the approximation is quite effective (Heckerman et al., 2000; Neville and Jensen, 2007). In practice, we have previously observed that when the cycles are large, i.e., the size of the clique in the undirected graph, the approximation is quite robust (Natarajan et al., 2012; De Raedt et al., 2016).

With this insight, in the first step of cycle detection, we identify the short cycles. The intuition is that short cycles lead to larger approximations and removing them would render the product of conditionals closer to the true joint distribution. Once the shortest cycle is identified, the next step is identifying the edge to remove from this short cycle. For this purpose, we employ mutual information (MI). As a pre-processing step, we compute the MI between every pair of variables and sort them by the MI. We consider MI instead of conditional MI as one of our key goals is efficiency. Computing conditional MI requires us to condition on a large set of related variables in the DN. This requires both repeated computations and a large number of conditionals. Thus, first, we detect the smallest directed cycle. We then break the cycle by removing edges that are smaller than a predefined threshold of δ. In our work, we simply choose δ to be the MI with the largest difference to the previous MI value in the sorted list. We use *Maximum adjacent difference* in the sorted list, as our δ in our setting, unless a default value is presented by an expert as domain knowledge. Large values of δ would result in a sparse graph and lower values δ will result in a dense graph. Once these edges are removed, the process continues where the next smallest cycle (if one exists) is detected and the low MI edges are removed and so on. **Coupling CSI with MI between variables** *X* **and** *Y* **quantifies the strength of** *X* → *Y*.

To summarize, from the DN, we create an initial CBN by detecting cycles and removing edges with low dependencies. Now the last step is to orient the bi-directed edges which are undirected and then learn the parameters of the resulting causal BN.

### 3.3. Edge Orientation and Parameter Learning

Once the directed cycles are detected and removed, we focus on the undirected edges (in reality bi-directed edges). Inspired by the PC algorithm (Spirtes et al., 2000), we orient the edges in the final step using two criteria—MI and acyclicity. We orient the edges by removing the edge with the lowest MI if it does not result in a cycle. As mentioned earlier, this is similar to that of PC. After all the undirected edges have been oriented, the resulting CBN is our casual network skeleton.

We estimate the parameters of this CBN using standard MLE (Pearl, 1988a). All our data sets are fully observed and hence MLE suffices for learning the conditional distributions. For the parameters, we learn a decision tree locally and in parallel using only the variables in the parent set of every node to capture the conditional distribution. Extending this to handle missing data is a significant extension as it does not merely affect the parameter learning but the structure search as well. Once the parameters are learned, we now have the full causal BN learned from data.

### 3.4. DN2CN Algorithm

**Figure 2:**
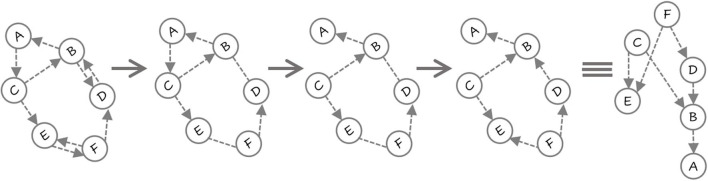
First the DN is learned (notice the two bi-directed edges). All the bidirected edges in the DN are converted to undirected edges (BD and EF). The shorted cycle *A* → *C* → *B* → *A* is identified and the edge *A* → *C* is removed based on MI. Since no more cycles exist, the undirected edges are considered next. *E* − − *F* becomes *F* → *E* and then *B* − − *D* becomes *D* → *B*. The resulting network is acylic and exploits both CSI and MI in becoming a causal network.

Before we provide the algorithm, we present an example in Figure 2. There are six variables 〈*A*, …, *F*〉. First, a DN is learned where there are cycles and bi-directed edges. Next, the smallest cycle 〈*A, B, C*〉 is detected and the edge with least MI *A* → *C* is removed. Now, there are no directed cycles in the CBN (in the general case, there could be more cycles that need to be removed). Note that there are two undirected edges between *B* and *D*, and between *E* and *F*. First, the edge between *D* and *B* is oriented based on MI and the fact that this does not create a cycle. Finally, the edge between *E* and *F* is oriented to obtain the CBN. The parameters are then learned by learning a decision-tree for each conditional.

This approach is formally presented in Algorithm 1 and as a flow chart in Figure 1. As can be seen in the algorithm, the first step is to learn the DN (line 4). The LearnParentSet function in line 3 of Algorithm 2 learns a tree and collects the set of parents from that set. It can optionally take an ordering among the variables provided by a domain expert (if any). Then the algorithm computes the mutual information (MI) for all the edges. One could instead simply wait till the cycles are detected and then compute the MI but we compute it outside the cycle detection step. The algorithm then iteratively removes the least informative edges till no more cycles are present in the graph. We orient the undirected edges (If any) ensuring acyclicity. Then the parameters are then learned from the data.

**Algorithm 1:** DN2CN: dependency network to causal network.

1: **Given**: Data **D**; Variables **V**; Ordering among variables (if any) *O*: = ∅; Threshold δ: = 0
2: **function** DN2CN(**D**,**V**, **O)**
3: **E**←∅ ⊳ Initialize edge set
4: DN ≡ (**V**, **E**) = **LearnDN**(**D**, **V**, *O*)
5: **for all** edge ∈ **E do**
6: MI[edge] ← ComputeMutualInfo(edge)
7: **end for**
8: SortedMI[edge] ← Sorted(edge, *reverse* = *True*) ⊳ **Sort in descending order**
9: **if** δ = 0 **then**
10: δ = argmax_AbsDiff(SortedMI[edge]) ⊳ **Max absolute diff of 2 contiguous elements in array SortedMI**
11: **end if**
12: **C** ← DetectCycles(DN) ⊳ **Using any sort**
13: **for all** cycle ∈ **C do**
14: **for all** *e* ∈ **cycle do**
15: **if** S*ortedMI*[*e*] ≤ δ **then**
16: **E**←**E**\*e* ⊳ **Remove edges if exist in DN**
17: **end if**
18: **end for**
19: **C**←**C**\cycle
20: ⊳ **Update cycles list after each iteration**
21: **if C** = ∅ **then** ⊳ **No more cycles left**
22: **break**
23: **end if**
24: **end for**
25: $\hat{\text{V}},\hat{\text{E}}:=\text{O}\text{RIENT}\text{E}\text{DGES}\left(\text{V},\text{E}\right)$ ⊳ **Introduce directions ensuring acyclicity as required**
26: **return** ($\hat{\text{V}},\hat{\text{E}}$**)**
27: **end function**

**Algorithm 2:** LearnDN: learn dependency network.

1: **function** LearnDN(**D**, **V**, **O)**
2: **E**←∅ ⊳ Initialize edge set
3: **for all** var ∈ **V do**
4: **P**(var) ← LearnParentSet(var, {**V**\var}_*O*_, **D**) ⊳ **Parent set** {**V**\var} **is constrained by O (if any)**
5: **for all** parent ∈ **P**(var) **do**
6: **E** ← **E** ∪ {parent → var}
7: ⊳ **Add new directed edge between parent and var**
8: **end for**
9: **end for**
10: **return** (**V**, **E**)
11: **end function**

#### 3.4.1. Theoretical Analysis

A natural question to ask is—*what is the complexity of our approach?* We present an initial analysis of this work, by adapting the arguments from the literature [see for instance the original reducibility result (Karp, 1972)]. We present our result by analyzing each component of the algorithm. Tightening these bounds with appropriate heuristics is left for future work.

Let *v* be the number of vertices (features), *n* be the number of training examples. In Algorithm 1, while learning *DN*, we learn a decision tree locally [line 4]. This requires *O*(*n*^2^*d*) where *d* is the depth of the tree (Su and Zhang, 2006). While this can be reduced to *O*(*n* · *d*), this requires making independence assumptions among the variables. Our tree growing procedure is fairly standard without much optimization. Hence the complexity of learning a full DN is *O*(*v* · *n*^2^*d*). However, the trees can be learned in parallel, thus reducing the complexity to *O*(*n*^2^*d*).

Cycle detection (line-12) has a complexity of *O*(*v*(*v* + *e*)), where *v* is no. of nodes and *e* is number of edges in the network (*e* is asymptotically *O*(*v*^2^). A single cycle detection running a DFS to search for the cycle thus is *O*(*v*^2^). Doing this for all the variables will result in *O*(*v*^3^) for the entire cycle detection. Sorting the edges to compute the MI requires *O*(*v*^2^*log*(*v*)). Edge orientation is *O*(*v*^2^).

Thus the complexity DN2CN is dominated by two terms—*O*(*v*^3^) the cube of the number of edges and *O*(*n*^2^*d*), the term that depends on the data. Since, typically, *n* > *v*^2^ to learn a meaningful model, our final complexity is *O*(*n*^2^*d*). Optimizing the tree learner to lower this complexity and better cycle detection methods to reduce the cubic complexity can significantly improve the asymptotic bound. These are open research directions.

#### 3.4.2. Discussion

The proposed approach has some salient advantages—(1) One could parallelize the learning of the DN to scale it up to very large data sets. (2) The computation of the MI can also be parallelized. (3) Any traversal algorithm could be used to detect cycles in the graph for pruning. (4) There are two levels of independence used in this algorithm;—(a) context specific independence (CSI) to identify potentially independent influences. Inspired by the work of Tikka et al. (2019), we rely on the ability of CSI to model interventions; in the context of interventions, any influences that otherwise have a causal effect thereon variable, are removed. Learning a BN as a series of trees for every interacting variable facilitates the ability to model such CSI and so are able to represent interventions in sufficient detail to reason about conditional independence properties, (b) Mutual independence which when combined with expert domain knowledge can potentially yield even causal influences. (5) The algorithm also has two types of controls (similar to regularizations) to combat overfitting. First is to control the depth of trees and second is selecting the number of edges to remove. (6) Finally, the use of both local search and constraint based methods inside the algorithm enables it to learn effectively at scale.

Before presenting our empirical results, we briefly discuss the interpretability of the resulting network. DN2CN represents causal dependencies using BNs that provide an intuitive visualization by modeling features as nodes and the statistical association between the features as edges. This statistical interpretability is similar in spirit to traditional interpretability. This allows to answer questions, such as “does BMI influence susceptibility to Covid?” Moreover, it has been argued that developing an effective CBN for practical applications requires expert knowledge when data collection is cumbersome (Fenton and Neil, 2012). This applies to domains, such as medicine, similar to our experimental evaluation. A typical characteristic of these domains is that they can be data-poor and knowledge-rich due to several decades of research. Kahneman et al. showed that human beings tend to interpret events in terms of cause-effect relations (Kahneman et al., 1982; Pennington and Hastie, 1988). Also, causal models are easier to construct, easier to modify and easier to interpret by humans (Henrion, 1987; Pennington and Hastie, 1988). Following these observations, our framework can incorporate both data-driven and human inputs, thus allowing to learn a more robust hypothesis. Lipton explains that with interpretable models it becomes imperative to guarantee fairness (Lipton, 2018). It must be noted that we can extend DN2CN's interactive framework and leverage the Bayesian networks learnt to assess the bias as well as compare multiple models in terms of their fairness and performance (Chiappa and Isaac, 2018). In summary, our framework can leverage interpretability as a tool to verify causal assumptions and relationships. We verify the above claims empirically in a real data set and two synthetic benchmark causal data sets in the next section.

## 4. Empirical Evaluation—Domains

To assess the effectiveness of our method, we perform extensive evaluations on both synthetic as well as real benchmark causal data sets. In all our data sets, we have the underlying true causal graph, and we apply our method as well baseline approaches to reconstruct the causal network from the data to demonstrate the effectiveness. We first describe the data sets used before discussing the baselines used.

### 4.1. Benchmark1: LUCAS—(LUng CAncer Simple Data Set)

The LUCAS (LUng CAncer Simple set) data set from causality challenge (Guyon et al., 2008) represents a synthetic medical diagnosis problem, where the task is to identify patients with lung cancer given a set of socioeconomic and clinical factors of putative causal relevance. The generative model is a Markov process, so the value of the children node is stochastically dependent on the values of the parent nodes'. The data set consists of 2000 observations. Ground-truth consists of 12 binary variables that include *anxiety, peer pressure, day of birth, smoking, genetics, yellow finger, lung cancer, attention disorder, cough, fatigue, allergy, car accidents*, and their causal relations. There are no missing values in the data set. As the data are generated artificially by causal BN with variables, the true nature of the underlying causal relationships is known. Hence we use this benchmark data set for illustrating the effectiveness of our approach.

### 4.2. Benchmark2: Asia Data Set

The ASIA Network is an expert-designed causal network with logical links. This BN was originally presented by Lauritzen and Spiegelhalter (Lauritzen and Spiegelhalter, 1988), who have specified reasonable transition properties for each variable given its parents. It is an eight node BN that describes the effect of visiting Asia and smoking behavior of an individual on the probability of contracting tuberculosis, cancer or bronchitis. The underlying structure expresses the known qualitative medical knowledge. Each node in the network represents a feature that relates to the patient's condition. The example is motivated as follows: “*Shortness-of-breath (called dyspnea) may be due to tuberculosis, lung cancer or bronchitis, or none of them, or more than one of them. A recent visit to Asia increases the chances of tuberculosis, while smoking is known to be a risk factor for both lung cancer and bronchitis. The results of a single chest X-ray do not discriminate between lung cancer and tuberculosis, as neither does the presence or absence of dyspnea.”* The data set contains 10,000 observations and eight binary variables whose values are 0 or 1. There are no missing values in the data set.

### 4.3. Causal Protein-Signaling Networks in Human T Cells Data Set

This data analyzed and published by Sachs et al. (2005) is a multivariate proteomics data set, widely used for research on causal discovery methods. This is a biological dataset with different proteins and phospholipids in human immune system cells. The data comprises of the simultaneous measurements of 11 phosphorylated proteins and phospholipids (PKC, PKA, P38, Jnk, Raf, Mek, Erk, Akt, Plcg, PIP2, PIP3) derived from thousands of individual primary immune system cells. In the data set we considered, there are (1) 1,800 observational data points subject only to general stimulatory cues, so that the protein signaling paths are active; (2) 600 interventional data points with specific stimulatory and inhibitory cues for each of the following four proteins: pmek, PIP2, Akt, PKA; and (3) 1,200 interventional data points with specific cues for PKA. Overall, the data set consists of 5,400 instances with no missing value. The 11 variables are discretized into three bins (low, medium, and high) for each feature, respectively. A network consisting of 18 well-established causal interactions between these molecules has been constructed supported with biological experiments and literature (Sachs et al., 2005). This data is a good fit to test our proposed causal discovery method, as the knowledge about the “ground truth” is available, which helps verification of results. Hence the goal of the data set is to unearth protein signaling networks, originally modeled as CBN.

## 5. Experimental Results

In our experiments, we aim to answer the following questions explicitly:

**Q1**: Does the learned model identify influencing variables as in the “Ground truth” network?
**Q2**: How does the resulting network produced by DN2CN compare to standard constraint based approaches qualitatively?
**Q3**: How does the resulting network produced by DN2CN compare to standard constraint based approaches quantitatively?

Specifically, we consider two different types of experiments—the first on evaluating **goodness** of the model on the synthetic benchmark data sets and the second on **verifying** if the approach can learn a good causal model on the real data set.

### 5.1. Setup

In DN2CN, we used a tree depth of 2 for all the experiments. We set δ as 0.015 for both LUCAS and Asia data sets and 0.25 for the real T cells data set.

We compare DN2CN to three of the well-known computational methods for causal discovery (Glymour et al., 2019). Two of these algorithms are commonly employed constraint-based algorithms—PC and Fast Causal Inference (FCI) (Spirtes et al., 2000). The third algorithm is a score-based algorithm—Fast Greedy Equivalence Search (FGES) (Ramsey et al., 2017). It must be mentioned that PC, FCI and FGES, are widely applicable as they handle various types of data distributions as well as causal relations, given reliable conditional independence testing methods. We strongly believe that these attributes make them a strong as well as a fair baseline for DN2CN as suggested by Glymour et al. (2019).

We further discuss each of the baseline approaches and their corresponding experimental settings used, as follows:

- *PC algorithm* (denoted **PC**) (Spirtes et al., 2000) starts with a fully connected undirected graph, tests all possible conditioning set for every order of conditioning and then finally orients the edges. Test statistic we used is the mutual information for PC algorithm, to keep the comparison fair. We used type I error rate; α = 0.05 in our setting.
- *Fast Greedy Equivalence Search algorithm* (denoted **FGES**) (Ramsey et al., 2017) is an optimized and parallelized version of an algorithm developed by Meek (Meek, 1995) called the Greedy Equivalence Search (GES). GES is a CBN learning algorithm that starts with an empty graph, heuristically performs a forward stepping search over the space of CBNs and stops with the one with the highest score. GES finally performs a backward stepping search that iteratively removes edges until no single edge removal can increase the Bayesian score. We use the modified BIC (Bayesian information criterion) (Schwarz, 1978) score rewritten as $Score_{BIC}\left(B:D\right)=2L\left(D;\hat{\theta },B\right)-k\log|D|$, where *L* is the likelihood, *k* the number of parameters, and |*D*| the sample size. So higher BIC scores will correspond to greater dependence.
- *Fast Causal Inference algorithm* (denoted **FCI**) (Spirtes et al., 2000) is a constraint-based algorithm which learns an equivalence class of CBNs that entail the set of conditional independencies that are true in the data. FCI then orients the edges using the stored conditioning sets that led to the removal of adjacencies earlier. We use the same modified BIC score as with the other baseline, i.e., FGES algorithm.

For PC algorithm we used the open-source implementation, i.e., *stable-PC* in bnlearn (Scutari, 2009) while TETRAD (Spirtes et al., 2000) was used to run FGES and FCI algorithms; a reliable tool for causal explorations. Data set details are presented in section 3 which describes the number of variables and the number of training examples.

### 5.2. Results

Recall that our goal is faithful modeling of underlying data. In addition, we also demonstrate the training log-likelihood of the learned model for (1) ground truth model, (2) model learnt using DN2CN algorithm, (3) model learnt using PC algorithm, (4) model learnt using FGES algorithm, and (5) model learnt using the FCI algorithm. This is to say that our analysis is *qualitative* as well as *quantitative*.

**Figure 3:**
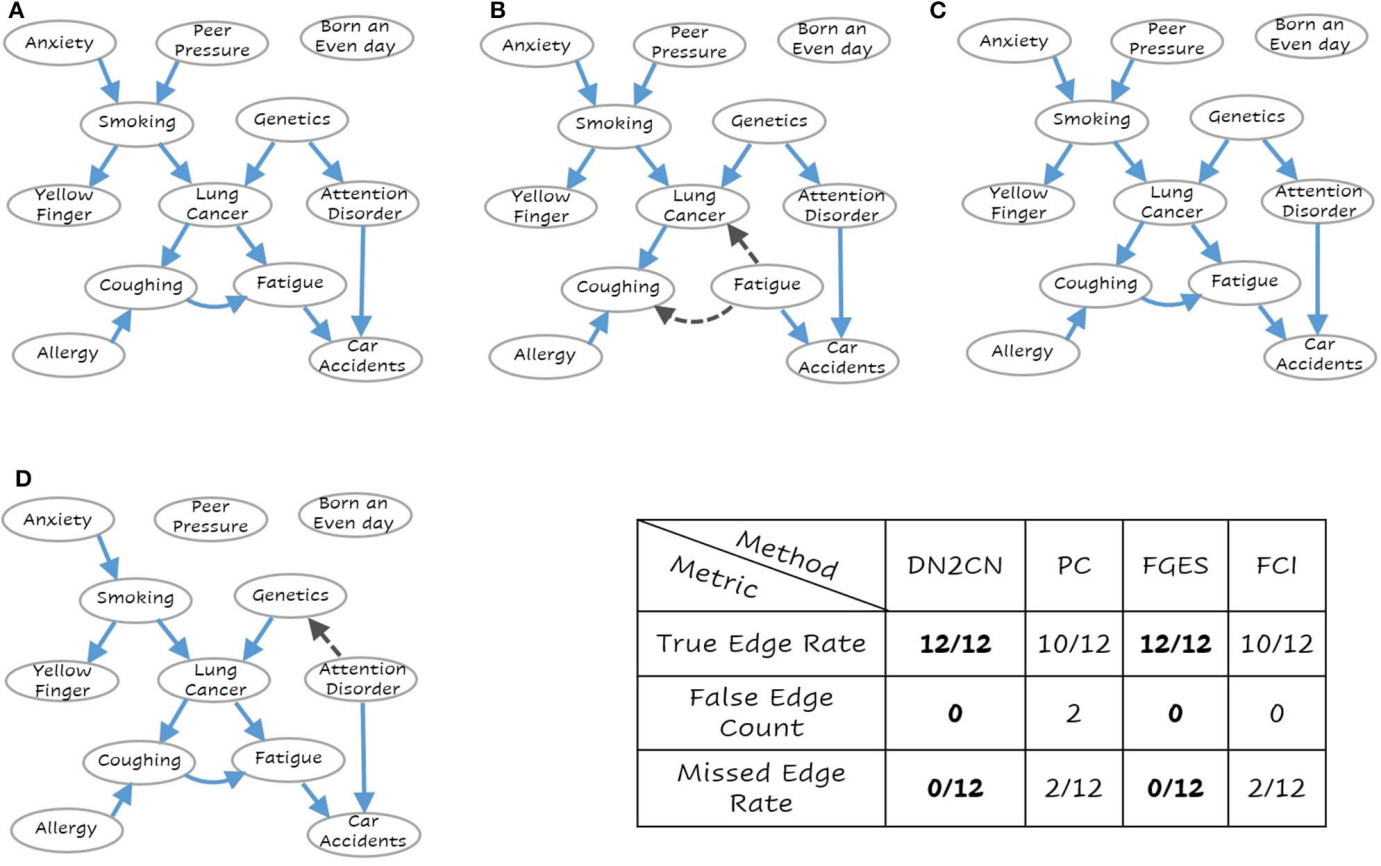
The learned network for **(A)** Our Approach DN2CN, **(B)** PC algorithm, **(C)** Fast Greedy Equivalence Search algorithm (FGES), and **(D)** Fast Causal Inference algorithm (FCI) and the summary results on LUCAS data set (best viewed in color). Each node represents a feature and the arcs represent causal relationships, i.e., X → Y represents that X is a cause of Y. As can be seen, our DN2CN and FGES had a 100% true positive rate with a 0 false positive and false negative rates. PC and FCI missed two edges each. PC and FCI also introduced spurious edges (incorrect edge orientation).

**Figure 4:**
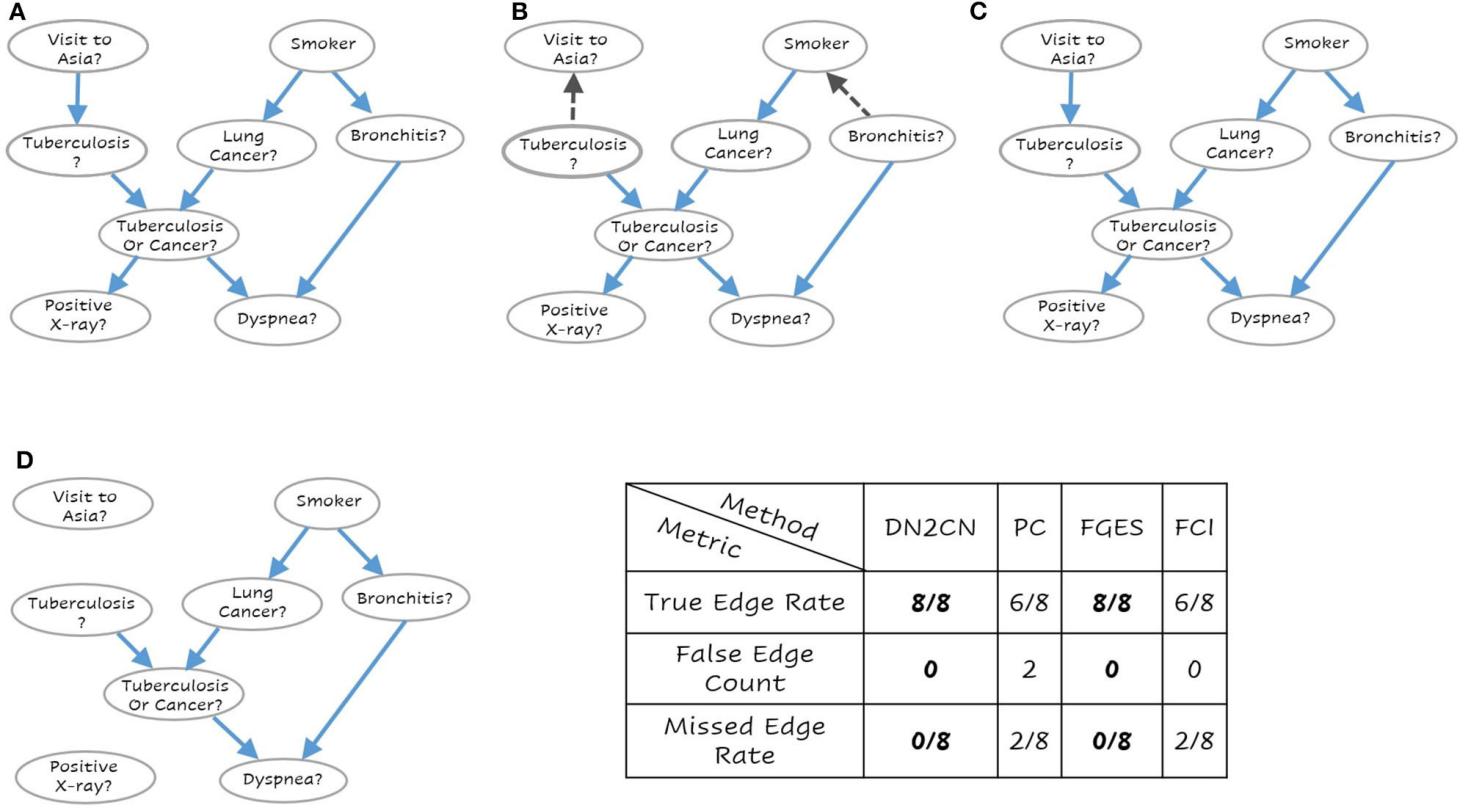
The learned network for **(A)** Our Approach DN2CN, **(B)** PC algorithm **(C)** Fast Greedy Equivalence Search algorithm (FGES), and **(D)** Fast Causal Inference algorithm (FCI) and the summary results on ASIA data set (best viewed in color). Each node represents a feature and the arcs represent causal relationships, i.e., X → Y represents that X is a cause of Y. As can be seen, our DN2CN and FGES had a 100% true positive rate with a 0 false positive and false negative rates. PC and FCI both missed two edges. Also, PC introduced two spurious causal edges in the resultant network.

**Figure 5:**
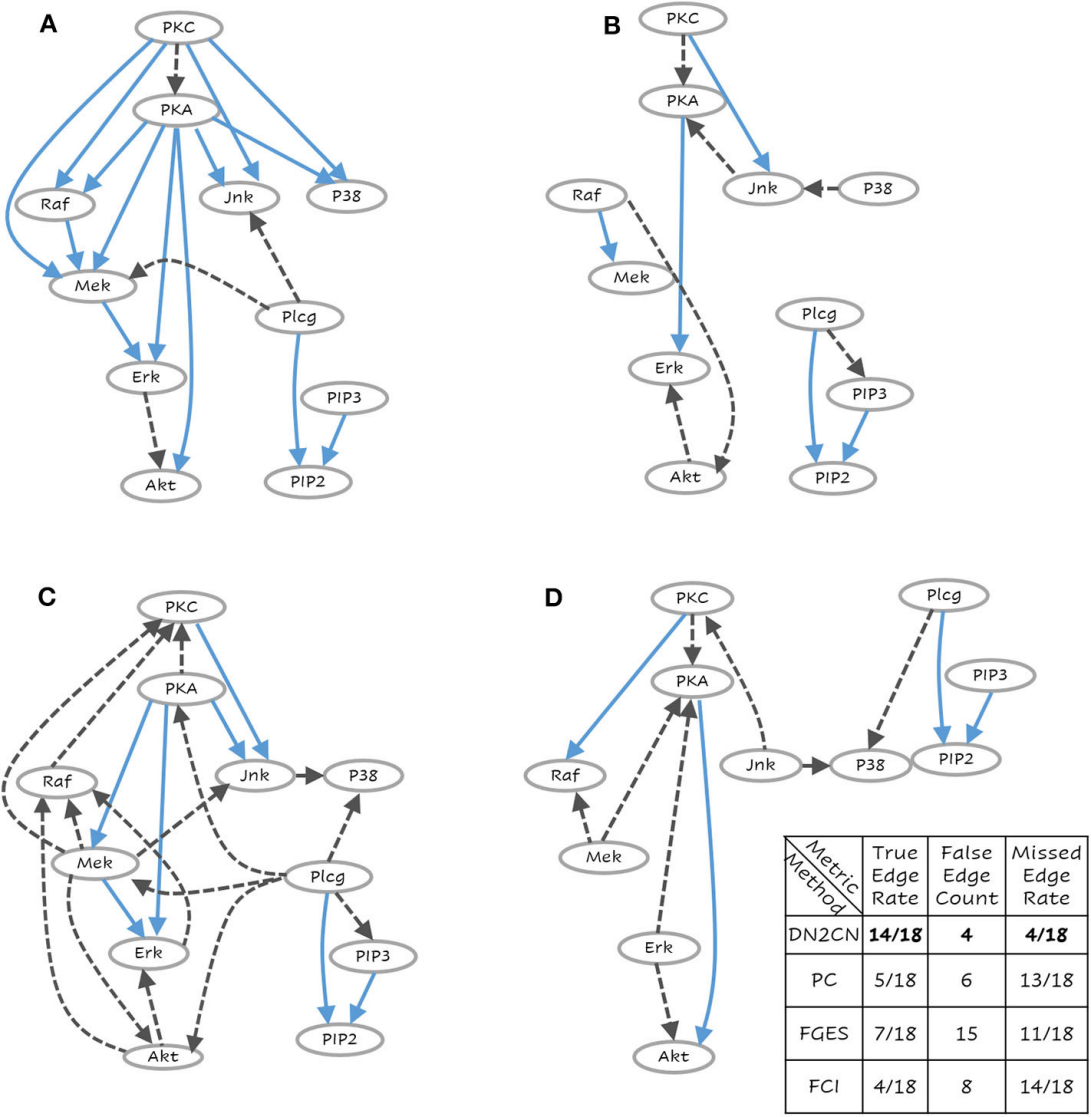
The learned network for **(A)** Our Approach DN2CN, **(B)** PC algorithm, **(C)** Fast Greedy Equivalence Search algorithm (FGES), and **(D)** Fast Causal Inference algorithm (FCI) and the summary results on T-Cell data set (best viewed in color). Each node represents a feature and the arcs represent causal relationships, i.e., X → Y represents that X is a cause of Y. This is a challenging data set where DN2CN had missed one edge and introduced two spurious edges. PC, on the other hand, had significantly worse performance with 10 missed edges and four spurious ones.

To answer **Q1 and Q2**, consider the networks presented in Figures 3A–D–5A–D, respectively. These are the learned networks obtained by our approach DN2CN and baseline methods PC, FGES & FCI summarized together with the ground truth network. To evaluate the validity of the proposed approach, we compared the model arcs with those present in the ground truth. An arc is correct, if and only if the same arc exists in the ground truth graph and the orientation of the arc aligns with the orientation in the ground truth graph; an arc is considered incorrect, if the arc does not exist in the ground truth graph or if it exists but its orientation is the opposite of the true orientation. Hence, in all the data sets, to understand the effectiveness of DN2CN, motivated by Sachs et al. (2005), Gao and Ji (2015), and Yu et al. (2019) we summarize the arcs learned by our method as well as PC, FGES and FCI for each data set using the following metrics:

- *True Edge Rate*, is the fraction of the true connections in the ground truth network that our approach (or PC or FGES or FCI) captures correctly, i.e., true positive.
- *False Edge Count*, for connections that are not in the ground truth network, but which were captured by our approach (or PC or FGES or FCI), i.e., false positive.
- *Missed Edge Rate*, is the fraction of the true edges missed in the ground network by our approach (or PC or FGES or FCI), i.e., a false negative.

As can be observed our algorithm DN2CN and baseline algorithm FGES had a 100% true positive rate with a 0 false positive and false negative rates in both LUCAS and ASIA data sets. However, the other baselines methods PC and FCI both missed two edges in LUCAS as well as ASIA data sets. In addition, the PC algorithm introduced spurious causal flows in both LUCAS and ASIA data sets. This clearly establishes that our framework is indeed capable of retrieving the full causal model while learning only from the data.

In the real benchmark data set, i.e., *Causal Protein-Signaling Network in human T cells*, the ground truth network and the reconstruction by employing DN2CN, PC, FGES and FCI are illustrated in Figures 5A–D, respectively. It can be observed that our approach DN2CN performs **significantly better** than all the baselines, i.e., PC, FGES and FCI. DN2CN missed four edges and introduced four spurious edges. Whereas, the baseline algorithms PC, FGES, and FCI, had significantly worse performance with 13, 11, 14 missed edges and 6, 15, 8 spurious ones, respectively. On closer inspection at the unexpected edges in our acyclic causal model reconstruction, one can see that they actually explain the data quite well. Especially, both arcs, PKC ⇒ PKA and Erk ⇒ Akt, can be understood qualitatively in rat ventricular myocytes (Wilhelm et al., 1997) and colon cancer cell lines (Lemaire et al., 1997), respectively. However, We hypothesize that, our DN2CN method missed four causal relationships, that are all involved in cycles. As BNs are acyclic by definition, our inference missed these arcs, which is one of the caveats of this approach. Extending this to dynamic causal bayesian network to handle feedback loops, remains an interesting future research direction.

Table 1 presents quantitative comparisons between the different methods. In all our experiments, we present the numbers in bold whenever they are better than all the other baselines on a data set. It must be mentioned that in some cases, PC, FGES, and FCI did not yield a directed arc, and we chose a direction (ensuring acyclicity) to compute the overall joint log-likelihood on the training set. As can be seen from the table, the proposed DN2CN approach produces a network with significantly better joint log-likelihood on the training set than the baseline algorithms PC and FCI learning method in all the domains. We can see that FGES has better joint log-likelihood than DN2CN in T-Cell data set. One key reason is that the resultant network using FGES is relatively denser than other models. FGES introduces 14 spurious causal edges leading to increased likelihood. It is well-known in the Bayes net learning literature that denser the graph is, higher the training set likelihood. As can be seen from the table in the Figure 5, the false edge count of FGES is significantly higher than the other methods. Hence, the denser network can yield a much higher training set loglikelihood. This answers **Q3** affirmatively: that DN2CN is more effective in modeling than the causal method, such as PC, FGES, and FCI.

**Table 1:** Table comparing the log-likelihood estimate in CBN learned using DN2CN and baseline approach, i.e., PC algorithm, Fast Greedy Equivalence Search algorithm (FGES) and Fast Causal Inference algorithm (FCI) learned directly from data.

	**Methods**				
**Data sets**	**Ground truth**	**DN2CN**	**PC**	**FGES**	**FCI**
Lucas	**−12130.83**	**−12130.83**	−12178.59	**−12130.83**	−12161.49
Asia	**−22212.85**	**−22212.85**	**−22212.85**	**−22212.85**	−23747.1
Sachs	−38723.1	−38081.29	−41930.74	**−35782.43**	−40822.13

*Numbers are presented in bold text whenever they are better than all the other baselines on a data set*.

## 6. Conclusions

We introduced a scalable causal learning algorithm that is capable of exploiting two types of independencies—context-specific independence (CSI) and conditional independence (CI). To exploit CSI, we learn a single tree for each variable in the model. Each tree can locally model and capture the CSI. Next, we orient and remove edges from this potentially cyclic model by computing the mutual information which allows for capturing the CIs. The intuition is that these two independence metrics have previously been explored in the context of causal learning and combining them will allow for learning a robust causal model. Our empirical evaluations in the standard data sets clearly demonstrate that the proposed DN2CN method does retrieve the true causal model in most of the domains. Most importantly, it does not introduce a denser model than what is necessary even if it means sacrificing the training likelihood. Thus, a natural regularization is achieved by controlling the depth of the trees and the orienting of edges as against other information-theoretic methods, such as BIC that employs a model complexity penalty.

There are several possible extensions of future work—adapting and applying these models to real problems in the lines of our previous work (Ramanan and Natarajan, 2019) is an important direction. Developing the theoretical underpinnings between CSI and CI with causal models is the next immediate direction. Converting the CSI from our models to do calculus and employing them in the context of learning from both observational and experimental data is another important problem. Finally, allowing for rich domain knowledge and inductive bias to guide the learner to a better causal model is possibly the most interesting direction.

## Data Availability Statement

The datasets analyzed for this study can be found in following repository, respectively: LUCAS—LUng CAncer Simple data set: http://www.causality.inf.ethz.ch/data/LUCAS.html; Asia data set: http://www.bnlearn.com/bnrepository/; Causal Protein-Signaling Networks in human T cells data set: http://www.bnlearn.com/bnrepository/.

## Author Contributions

NR and SN contributed equally to the ideation and contributed nearly equally to the manuscript preparation. NR led the empirical evaluation. All authors contributed to the article and approved the submitted version.

## Conflict of Interest

The authors declare that the research was conducted in the absence of any commercial or financial relationships that could be construed as a potential conflict of interest.
